# Bis[μ_2_-*N*′-acetyl-2-hydroxy-6-oxido­benzohydrazidato(3−)]octa­pyridine­trinickel(II)

**DOI:** 10.1107/S1600536809020534

**Published:** 2009-06-06

**Authors:** Yan Yang, Dacheng Li, Xuefeng Shi

**Affiliations:** a College of Chemistry and Chemical Engineering, Liaocheng University, Shandong 252059, People’s Republic of China

## Abstract

The title trinuclear complex, [Ni_3_(C_9_H_7_N_2_O_4_)_2_(C_5_H_5_N)_8_], possesses a crystallographically imposed center of symmetry occupied by one Ni^II^ ion. Each of the three Ni^II^ ions is coordinated by two O and four N atoms, respectively, in a distorted octa­hedral geometry. In the crystal, weak inter­molecular C—H⋯π inter­actions link the mol­ecules into ribbons propagating in the [100] direction.

## Related literature

For applications of *N*-acetyl­salicylhydrazide complexes, see: John *et al.* (2004 ▶); Lin *et al.* (2002 ▶).
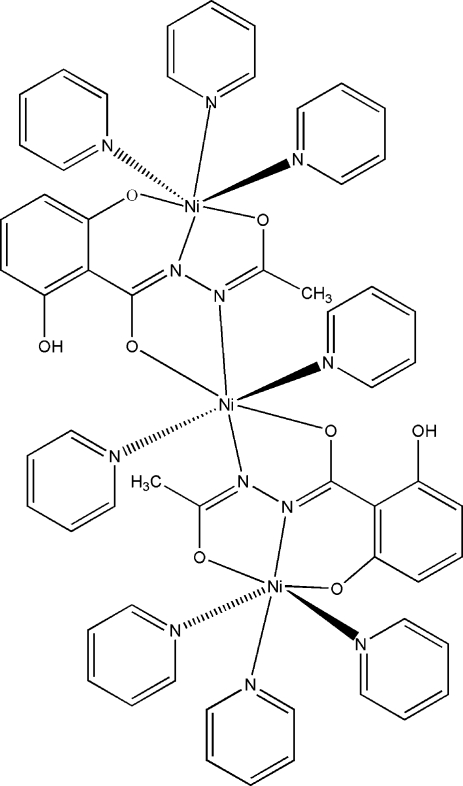


## Experimental

### 

#### Crystal data

[Ni_3_(C_9_H_7_N_2_O_4_)_2_(C_5_H_5_N)_8_]
*M* *_r_* = 1223.26
Monoclinic, 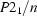

*a* = 9.9349 (18) Å
*b* = 18.230 (3) Å
*c* = 16.262 (2) Å
β = 96.663 (2)°
*V* = 2925.3 (8) Å^3^
*Z* = 2
Mo *K*α radiation
μ = 1.02 mm^−1^
*T* = 298 K
0.53 × 0.45 × 0.44 mm

#### Data collection

Bruker SMART CCD area-detector diffractometer
Absorption correction: multi-scan (*SADABS*; Sheldrick, 1996 ▶) *T*_min_ = 0.614, *T*_max_ = 0.663
14103 measured reflections
5138 independent reflections
3047 reflections with *I* > 2σ(*I*)
*R*_int_ = 0.146

#### Refinement

*R*[*F* ^2^ > 2σ(*F* ^2^)] = 0.075
*wR*(*F* ^2^) = 0.210
*S* = 0.97
5138 reflections
368 parameters
1098 restraints
H-atom parameters constrained
Δρ_max_ = 1.32 e Å^−3^
Δρ_min_ = −0.58 e Å^−3^

### 

Data collection: *SMART* (Siemens, 1996 ▶); cell refinement: *SAINT* (Siemens, 1996 ▶); data reduction: *SAINT*; program(s) used to solve structure: *SHELXS97* (Sheldrick, 2008 ▶); program(s) used to refine structure: *SHELXL97* (Sheldrick, 2008 ▶); molecular graphics: *SHELXTL* (Sheldrick, 2008 ▶); software used to prepare material for publication: *SHELXTL*.

## Supplementary Material

Crystal structure: contains datablocks I, global. DOI: 10.1107/S1600536809020534/cv2565sup1.cif

Structure factors: contains datablocks I. DOI: 10.1107/S1600536809020534/cv2565Isup2.hkl

Additional supplementary materials:  crystallographic information; 3D view; checkCIF report

## Figures and Tables

**Table 1:** Hydrogen-bond geometry (Å, °)

*D*—H⋯*A*	*D*—H	H⋯*A*	*D*⋯*A*	*D*—H⋯*A*
C27—H27⋯*Cg*^i^	0.93	2.52	3.428 (4)	166

Symmetry code: (i) 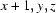. *Cg* is the centroid of the C2–C7 ring.

**Fig. 1.**
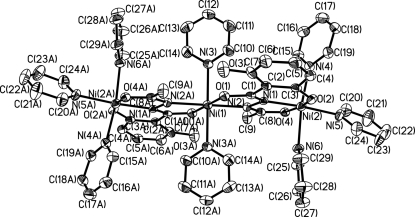
The molecular structure of the title complex, showing 40% probability displacement ellipsoids and the atomic numbering [symmetry code: (A) -x, -y, 2-z]. H atoms have been omitted for clarity.

**Fig. 2.**
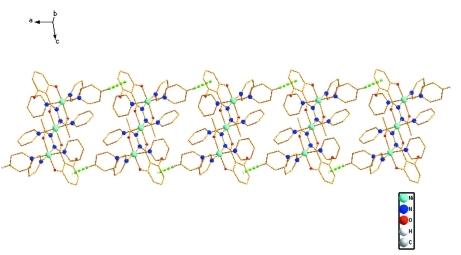
A portion of the crystal packing showing one-dimensional ribbons. Intermolecular C—H···π interactions are shown as dashed lines. Most of H atoms are omitted.

## supplementary crystallographic information

### Comment

In recent years, a large number of *N*-acetylsalicylhydrazide complexes have been prepared and studied due to their potential applications in chemically modified (Lin *et al.*, 2002) and anion-selective separation agents (John *et al.*, 2004). However, structures of nickel(II) complexes with *N*-acetyl-6-hydroxysalicylhydrzaide were not reported. So we have synthesized the title compound, which has been characterized by X-ray diffraction and elemental analysis.

The title complex (Fig. 1) contains three nickel(II) centers having distorted octahedral coordination environment, two ligand molecules and eight pyridine molecules. The triple-deprotonated *N*-acetyl-6-hydroxysalicylhydrzaide ligands bridge metal ions *via* hydrazide N—N group forming trinuclear nickel complex. In the crystal,  the complex molecules are linked into ribbons *via* intermolecular C—H···π weak interactions (Table 1, Fig. 2).

### Experimental

A solution of Ni(OAc)_2.4_H_2_O (0.4 mmol, 0.0996 g) in dimethylformamide (10 ml) was add to *N*-acetyl-(6-hydroxysalicylhydrzaide) (0.2 mmol, 0.042 g) in pyridine (10 ml). A reddish solution was obtained after refluxing for 3 h. After the solution had been stanging for two weeks, red block crystals suitable for X-ray diffraction appear.(m.p. >400 K). Elemental analysis calculated for C_58_H_54_N_12_O_8_Ni_3_: C 56.95, H 4.45, N 13.74%; found: C 56.92, H 4.44, N 13.76%.

### Refinement

All H atoms were placed geometrically and treated as riding on their parent atoms with C—H 0.93 (pyridine, benzene) or 0.97 (methylene) Å [*U*_iso_(H) = 1.2*U*_eq_(C)], and O—H 0.82 Å (hydroxyl) [*U*_iso_(H) = 1.5*U*_eq_(O)].

### Crystal data

**Table:**

[Ni_3_(C_9_H_7_N_2_O_4_)_2_(C_5_H_5_N)_8_]	*F*(000) = 1268
*M**_r_* = 1223.26	*D*_x_ = 1.389 Mg m^−^^3^
Monoclinic, *P*2_1_/*n*	Mo *K*α radiation, λ = 0.71073 Å
*a* = 9.9349 (18) Å	Cell parameters from 4241 reflections
*b* = 18.230 (3) Å	θ = 2.2–26.2°
*c* = 16.262 (2) Å	µ = 1.02 mm^−^^1^
β = 96.663 (2)°	*T* = 298 K
*V* = 2925.3 (8) Å^3^	Block, red
*Z* = 2	0.53 × 0.45 × 0.44 mm

### Data collection

**Table:**

Bruker SMART CCD area-detector diffractometer	5138 independent reflections
Radiation source: fine-focus sealed tube	3047 reflections with *I* > 2σ(*I*)
graphite	*R*_int_ = 0.146
phi and ω scans	θ_max_ = 25.0°, θ_min_ = 1.7°
Absorption correction: multi-scan (*SADABS*; Sheldrick, 1996)	*h* = −11→11
*T*_min_ = 0.614, *T*_max_ = 0.663	*k* = −16→21
14103 measured reflections	*l* = −19→19

### Refinement

**Table:**

Refinement on *F*^2^	Primary atom site location: structure-invariant direct methods
Least-squares matrix: full	Secondary atom site location: difference Fourier map
*R*[*F*^2^ > 2σ(*F*^2^)] = 0.075	Hydrogen site location: inferred from neighbouring sites
*wR*(*F*^2^) = 0.210	H-atom parameters constrained
*S* = 0.97	*w* = 1/[σ^2^(*F*_o_^2^) + (0.0999*P*)^2^] where *P* = (*F*_o_^2^ + 2*F*_c_^2^)/3
5138 reflections	(Δ/σ)_max_ < 0.001
368 parameters	Δρ_max_ = 1.32 e Å^−^^3^
1098 restraints	Δρ_min_ = −0.58 e Å^−^^3^

### Special details

**Table:**

Geometry. All e.s.d.'s (except the e.s.d. in the dihedral angle between two l.s. planes) are estimated using the full covariance matrix. The cell e.s.d.'s are taken into account individually in the estimation of e.s.d.'s in distances, angles and torsion angles; correlations between e.s.d.'s in cell parameters are only used when they are defined by crystal symmetry. An approximate (isotropic) treatment of cell e.s.d.'s is used for estimating e.s.d.'s involving l.s. planes.
Refinement. Refinement of *F*^2^ against ALL reflections. The weighted *R*-factor *wR* and goodness of fit *S* are based on *F*^2^, conventional *R*-factors *R* are based on *F*, with *F* set to zero for negative *F*^2^. The threshold expression of *F*^2^ > σ(*F*^2^) is used only for calculating *R*-factors(gt) *etc*. and is not relevant to the choice of reflections for refinement. *R*-factors based on *F*^2^ are statistically about twice as large as those based on *F*, and *R*- factors based on ALL data will be even larger.

### Fractional atomic coordinates and isotropic or equivalent isotropic displacement parameters (Å^2^)

**Table:**

	*x*	*y*	*z*	*U*_iso_*/*U*_eq_	
Ni1	0.0000	0.0000	1.0000	0.0357 (3)	
Ni2	−0.07930 (7)	0.24578 (3)	0.88362 (4)	0.0416 (3)	
N1	−0.0186 (4)	0.1420 (2)	0.9063 (2)	0.0332 (9)	
N2	−0.0473 (4)	0.11377 (19)	0.9860 (2)	0.0338 (9)	
N3	0.2129 (4)	0.0283 (2)	1.0484 (3)	0.0434 (10)	
N4	0.1185 (5)	0.2968 (2)	0.9214 (3)	0.0502 (11)	
N5	−0.1604 (5)	0.3551 (2)	0.8698 (3)	0.0525 (11)	
N6	−0.2903 (4)	0.2099 (2)	0.8443 (3)	0.0506 (11)	
O1	0.0447 (3)	0.02165 (18)	0.88104 (19)	0.0391 (8)	
O2	−0.0350 (4)	0.23782 (18)	0.7661 (2)	0.0441 (8)	
O3	0.1899 (5)	−0.0024 (2)	0.7669 (3)	0.0712 (14)	
H3	0.1430	−0.0122	0.8037	0.107*	
O4	−0.1147 (3)	0.23505 (17)	1.0063 (2)	0.0395 (8)	
C1	0.0280 (5)	0.0917 (3)	0.8579 (3)	0.0338 (11)	
C2	0.0716 (5)	0.1143 (3)	0.7758 (3)	0.0398 (11)	
C3	0.0407 (5)	0.1859 (3)	0.7368 (3)	0.0411 (11)	
C4	0.0967 (5)	0.2028 (3)	0.6618 (3)	0.0473 (13)	
H4	0.0820	0.2490	0.6381	0.057*	
C5	0.1731 (6)	0.1513 (3)	0.6234 (3)	0.0544 (15)	
H5	0.2057	0.1631	0.5737	0.065*	
C6	0.2008 (6)	0.0828 (3)	0.6585 (3)	0.0566 (15)	
H6	0.2515	0.0490	0.6323	0.068*	
C7	0.1524 (6)	0.0643 (3)	0.7334 (3)	0.0472 (13)	
C8	−0.0932 (5)	0.1671 (3)	1.0307 (3)	0.0398 (11)	
C9	−0.1235 (7)	0.1487 (3)	1.1180 (4)	0.0637 (18)	
H9A	−0.1324	0.1933	1.1484	0.096*	
H9B	−0.0508	0.1199	1.1454	0.096*	
H9C	−0.2065	0.1213	1.1153	0.096*	
C10	0.2673 (8)	0.0927 (4)	1.0400 (5)	0.0862 (19)	
H10	0.2164	0.1269	1.0074	0.103*	
C11	0.3967 (9)	0.1145 (4)	1.0763 (6)	0.107 (3)	
H11	0.4251	0.1627	1.0710	0.129*	
C12	0.4777 (7)	0.0663 (4)	1.1179 (5)	0.088 (2)	
H12	0.5658	0.0786	1.1390	0.105*	
C13	0.4269 (8)	−0.0020 (5)	1.1287 (6)	0.107 (3)	
H13	0.4792	−0.0373	1.1589	0.129*	
C14	0.2941 (8)	−0.0185 (4)	1.0936 (5)	0.090 (2)	
H14	0.2607	−0.0651	1.1025	0.108*	
C15	0.1824 (6)	0.2935 (3)	0.9993 (4)	0.0557 (14)	
H15	0.1414	0.2668	1.0382	0.067*	
C16	0.3044 (7)	0.3272 (3)	1.0250 (4)	0.0666 (16)	
H16	0.3460	0.3219	1.0789	0.080*	
C17	0.3628 (7)	0.3692 (4)	0.9674 (5)	0.0728 (18)	
H17	0.4438	0.3938	0.9829	0.087*	
C18	0.3011 (7)	0.3746 (3)	0.8871 (5)	0.0688 (17)	
H18	0.3398	0.4023	0.8480	0.083*	
C19	0.1795 (7)	0.3376 (3)	0.8662 (4)	0.0604 (15)	
H19	0.1379	0.3409	0.8120	0.073*	
C20	−0.1519 (7)	0.4020 (3)	0.9337 (4)	0.0663 (16)	
H20	−0.1007	0.3891	0.9831	0.080*	
C21	−0.2174 (9)	0.4691 (4)	0.9283 (5)	0.091 (2)	
H21	−0.2080	0.5009	0.9733	0.109*	
C22	−0.2966 (9)	0.4892 (4)	0.8563 (5)	0.088 (2)	
H22	−0.3422	0.5338	0.8527	0.106*	
C23	−0.3065 (8)	0.4426 (4)	0.7915 (5)	0.088 (2)	
H23	−0.3595	0.4543	0.7423	0.105*	
C24	−0.2355 (7)	0.3765 (3)	0.7999 (4)	0.0719 (17)	
H24	−0.2404	0.3454	0.7543	0.086*	
C25	−0.3903 (6)	0.2194 (3)	0.8929 (4)	0.0618 (15)	
H25	−0.3665	0.2354	0.9470	0.074*	
C26	−0.5244 (7)	0.2068 (4)	0.8675 (5)	0.0719 (18)	
H26	−0.5888	0.2123	0.9042	0.086*	
C27	−0.5625 (7)	0.1861 (4)	0.7873 (5)	0.0825 (19)	
H27	−0.6535	0.1789	0.7683	0.099*	
C28	−0.4647 (7)	0.1759 (4)	0.7351 (5)	0.086 (2)	
H28	−0.4876	0.1617	0.6803	0.103*	
C29	−0.3281 (7)	0.1878 (4)	0.7675 (4)	0.0682 (16)	
H29	−0.2612	0.1797	0.7330	0.082*	

### Atomic displacement parameters (Å^2^)

**Table:**

	*U*^11^	*U*^22^	*U*^33^	*U*^12^	*U*^13^	*U*^23^
Ni1	0.0354 (5)	0.0393 (5)	0.0350 (5)	−0.0006 (4)	0.0142 (4)	0.0001 (4)
Ni2	0.0364 (4)	0.0471 (4)	0.0422 (4)	0.0048 (3)	0.0081 (3)	0.0048 (3)
N1	0.026 (2)	0.0410 (19)	0.033 (2)	0.0001 (16)	0.0076 (17)	0.0064 (17)
N2	0.035 (2)	0.0358 (19)	0.032 (2)	0.0048 (17)	0.0112 (17)	0.0031 (16)
N3	0.039 (2)	0.047 (2)	0.046 (2)	−0.004 (2)	0.012 (2)	0.0028 (19)
N4	0.048 (3)	0.048 (2)	0.055 (3)	−0.002 (2)	0.008 (2)	0.005 (2)
N5	0.055 (3)	0.050 (2)	0.053 (3)	0.015 (2)	0.009 (2)	0.010 (2)
N6	0.036 (2)	0.058 (2)	0.058 (3)	0.001 (2)	0.009 (2)	−0.003 (2)
O1	0.040 (2)	0.0467 (18)	0.0337 (18)	−0.0003 (16)	0.0150 (16)	0.0000 (14)
O2	0.0369 (19)	0.0571 (19)	0.0390 (19)	0.0092 (16)	0.0071 (16)	0.0088 (15)
O3	0.101 (4)	0.057 (2)	0.066 (3)	0.011 (2)	0.052 (3)	0.003 (2)
O4	0.0376 (19)	0.0430 (17)	0.040 (2)	0.0016 (15)	0.0137 (16)	−0.0014 (15)
C1	0.025 (2)	0.043 (2)	0.035 (2)	−0.0048 (19)	0.008 (2)	−0.001 (2)
C2	0.036 (3)	0.050 (3)	0.036 (3)	−0.007 (2)	0.011 (2)	0.005 (2)
C3	0.032 (3)	0.053 (3)	0.040 (3)	−0.006 (2)	0.009 (2)	0.005 (2)
C4	0.043 (3)	0.058 (3)	0.041 (3)	0.000 (3)	0.006 (3)	0.009 (2)
C5	0.049 (3)	0.073 (3)	0.044 (3)	−0.007 (3)	0.016 (3)	0.011 (3)
C6	0.061 (4)	0.063 (3)	0.050 (3)	0.003 (3)	0.026 (3)	−0.004 (3)
C7	0.056 (3)	0.044 (3)	0.046 (3)	−0.010 (2)	0.018 (3)	0.001 (2)
C8	0.034 (3)	0.046 (3)	0.040 (3)	−0.004 (2)	0.009 (2)	0.000 (2)
C9	0.085 (5)	0.053 (3)	0.061 (4)	0.013 (3)	0.040 (4)	0.009 (3)
C10	0.065 (4)	0.075 (4)	0.110 (4)	−0.018 (3)	−0.025 (4)	0.030 (3)
C11	0.079 (5)	0.086 (5)	0.145 (6)	−0.032 (4)	−0.038 (5)	0.029 (4)
C12	0.053 (4)	0.085 (4)	0.120 (6)	−0.017 (4)	−0.014 (4)	0.018 (4)
C13	0.070 (5)	0.095 (5)	0.147 (7)	0.007 (4)	−0.027 (5)	0.022 (4)
C14	0.071 (4)	0.066 (4)	0.125 (5)	−0.008 (4)	−0.022 (4)	0.009 (4)
C15	0.050 (3)	0.060 (3)	0.057 (3)	−0.003 (3)	0.005 (3)	−0.001 (3)
C16	0.057 (4)	0.074 (4)	0.067 (4)	0.003 (3)	−0.001 (3)	−0.002 (3)
C17	0.055 (4)	0.071 (4)	0.093 (5)	−0.005 (3)	0.010 (4)	−0.010 (3)
C18	0.058 (4)	0.057 (3)	0.092 (5)	−0.007 (3)	0.016 (4)	0.011 (3)
C19	0.058 (3)	0.056 (3)	0.069 (4)	0.001 (3)	0.011 (3)	0.010 (3)
C20	0.075 (4)	0.066 (3)	0.060 (3)	0.013 (3)	0.018 (3)	0.005 (3)
C21	0.114 (5)	0.082 (4)	0.076 (5)	0.032 (4)	0.006 (4)	−0.004 (4)
C22	0.101 (5)	0.072 (4)	0.093 (5)	0.036 (4)	0.014 (4)	0.013 (4)
C23	0.096 (5)	0.090 (5)	0.074 (4)	0.032 (4)	−0.007 (4)	0.025 (4)
C24	0.080 (4)	0.066 (3)	0.068 (4)	0.006 (3)	0.003 (3)	0.002 (3)
C25	0.046 (3)	0.073 (3)	0.068 (4)	0.006 (3)	0.012 (3)	−0.007 (3)
C26	0.043 (3)	0.089 (4)	0.086 (5)	0.003 (3)	0.013 (3)	−0.007 (4)
C27	0.047 (4)	0.099 (5)	0.101 (5)	0.001 (4)	0.004 (4)	0.002 (4)
C28	0.055 (4)	0.122 (5)	0.078 (5)	−0.013 (4)	0.003 (4)	−0.002 (4)
C29	0.050 (3)	0.095 (4)	0.060 (3)	−0.007 (3)	0.006 (3)	−0.006 (3)

### Geometric parameters (Å, °)

**Table:**

Ni1—O1^i^	2.072 (3)	C9—H9A	0.9600
Ni1—O1	2.072 (3)	C9—H9B	0.9600
Ni1—N2	2.133 (4)	C9—H9C	0.9600
Ni1—N2^i^	2.133 (4)	C10—C11	1.408 (10)
Ni1—N3^i^	2.230 (4)	C10—H10	0.9300
Ni1—N3	2.230 (4)	C11—C12	1.323 (10)
Ni2—N1	2.007 (4)	C11—H11	0.9300
Ni2—O2	2.016 (3)	C12—C13	1.363 (10)
Ni2—O4	2.074 (3)	C12—H12	0.9300
Ni2—N5	2.152 (4)	C13—C14	1.407 (10)
Ni2—N4	2.196 (5)	C13—H13	0.9300
Ni2—N6	2.218 (5)	C14—H14	0.9300
N1—C1	1.326 (6)	C15—C16	1.379 (8)
N1—N2	1.453 (5)	C15—H15	0.9300
N2—C8	1.326 (6)	C16—C17	1.388 (8)
N3—C10	1.305 (7)	C16—H16	0.9300
N3—C14	1.335 (8)	C17—C18	1.380 (9)
N4—C15	1.351 (7)	C17—H17	0.9300
N4—C19	1.362 (7)	C18—C19	1.391 (9)
N5—C20	1.342 (7)	C18—H18	0.9300
N5—C24	1.343 (8)	C19—H19	0.9300
N6—C29	1.324 (7)	C20—C21	1.383 (9)
N6—C25	1.351 (6)	C20—H20	0.9300
O1—C1	1.336 (6)	C21—C22	1.382 (10)
O2—C3	1.331 (6)	C21—H21	0.9300
O3—C7	1.367 (6)	C22—C23	1.348 (10)
O3—H3	0.8200	C22—H22	0.9300
O4—C8	1.310 (6)	C23—C24	1.396 (9)
C1—C2	1.509 (6)	C23—H23	0.9300
C2—C7	1.442 (7)	C24—H24	0.9300
C2—C3	1.467 (7)	C25—C26	1.368 (9)
C3—C4	1.432 (7)	C25—H25	0.9300
C4—C5	1.399 (7)	C26—C27	1.369 (9)
C4—H4	0.9300	C26—H26	0.9300
C5—C6	1.387 (8)	C27—C28	1.375 (9)
C5—H5	0.9300	C27—H27	0.9300
C6—C7	1.401 (7)	C28—C29	1.414 (9)
C6—H6	0.9300	C28—H28	0.9300
C8—C9	1.523 (7)	C29—H29	0.9300
			
O1^i^—Ni1—O1	180.000 (1)	O3—C7—C2	120.9 (4)
O1^i^—Ni1—N2	102.49 (13)	C6—C7—C2	122.0 (5)
O1—Ni1—N2	77.51 (13)	O4—C8—N2	125.6 (4)
O1^i^—Ni1—N2^i^	77.51 (13)	O4—C8—C9	116.6 (4)
O1—Ni1—N2^i^	102.49 (13)	N2—C8—C9	117.8 (4)
N2—Ni1—N2^i^	180.000 (1)	C8—C9—H9A	109.5
O1^i^—Ni1—N3^i^	89.33 (14)	C8—C9—H9B	109.5
O1—Ni1—N3^i^	90.67 (14)	H9A—C9—H9B	109.5
N2—Ni1—N3^i^	89.97 (15)	C8—C9—H9C	109.5
N2^i^—Ni1—N3^i^	90.03 (16)	H9A—C9—H9C	109.5
O1^i^—Ni1—N3	90.67 (14)	H9B—C9—H9C	109.5
O1—Ni1—N3	89.33 (14)	N3—C10—C11	125.4 (7)
N2—Ni1—N3	90.03 (16)	N3—C10—H10	117.3
N2^i^—Ni1—N3	89.97 (15)	C11—C10—H10	117.3
N3^i^—Ni1—N3	180.0	C12—C11—C10	119.9 (7)
N1—Ni2—O2	90.71 (14)	C12—C11—H11	120.1
N1—Ni2—O4	79.35 (14)	C10—C11—H11	120.1
O2—Ni2—O4	170.05 (13)	C11—C12—C13	117.4 (7)
N1—Ni2—N5	173.36 (15)	C11—C12—H12	121.3
O2—Ni2—N5	95.04 (15)	C13—C12—H12	121.3
O4—Ni2—N5	94.86 (15)	C12—C13—C14	119.1 (8)
N1—Ni2—N4	96.18 (16)	C12—C13—H13	120.5
O2—Ni2—N4	90.50 (16)	C14—C13—H13	120.5
O4—Ni2—N4	90.97 (15)	N3—C14—C13	124.4 (7)
N5—Ni2—N4	87.10 (18)	N3—C14—H14	117.8
N1—Ni2—N6	91.67 (16)	C13—C14—H14	117.8
O2—Ni2—N6	90.48 (16)	N4—C15—C16	124.5 (6)
O4—Ni2—N6	89.41 (15)	N4—C15—H15	117.8
N5—Ni2—N6	84.98 (18)	C16—C15—H15	117.8
N4—Ni2—N6	172.08 (16)	C15—C16—C17	117.6 (7)
C1—N1—N2	113.7 (4)	C15—C16—H16	121.2
C1—N1—Ni2	131.4 (3)	C17—C16—H16	121.2
N2—N1—Ni2	114.3 (3)	C18—C17—C16	120.2 (7)
C8—N2—N1	110.1 (4)	C18—C17—H17	119.9
C8—N2—Ni1	137.8 (3)	C16—C17—H17	119.9
N1—N2—Ni1	112.1 (2)	C17—C18—C19	118.4 (6)
C10—N3—C14	113.6 (6)	C17—C18—H18	120.8
C10—N3—Ni1	124.0 (4)	C19—C18—H18	120.8
C14—N3—Ni1	122.3 (4)	N4—C19—C18	123.0 (6)
C15—N4—C19	116.4 (5)	N4—C19—H19	118.5
C15—N4—Ni2	123.5 (4)	C18—C19—H19	118.5
C19—N4—Ni2	120.0 (4)	N5—C20—C21	121.8 (6)
C20—N5—C24	116.8 (5)	N5—C20—H20	119.1
C20—N5—Ni2	121.3 (4)	C21—C20—H20	119.1
C24—N5—Ni2	121.5 (4)	C22—C21—C20	120.4 (7)
C29—N6—C25	116.4 (5)	C22—C21—H21	119.8
C29—N6—Ni2	121.1 (4)	C20—C21—H21	119.8
C25—N6—Ni2	121.9 (4)	C23—C22—C21	118.6 (7)
C1—O1—Ni1	114.3 (3)	C23—C22—H22	120.7
C3—O2—Ni2	125.8 (3)	C21—C22—H22	120.7
C7—O3—H3	109.5	C22—C23—C24	118.5 (7)
C8—O4—Ni2	109.9 (3)	C22—C23—H23	120.8
N1—C1—O1	122.3 (4)	C24—C23—H23	120.8
N1—C1—C2	119.5 (4)	N5—C24—C23	123.9 (6)
O1—C1—C2	118.1 (4)	N5—C24—H24	118.0
C7—C2—C3	117.1 (4)	C23—C24—H24	118.0
C7—C2—C1	118.9 (4)	N6—C25—C26	123.9 (6)
C3—C2—C1	123.9 (4)	N6—C25—H25	118.1
O2—C3—C4	116.3 (4)	C26—C25—H25	118.1
O2—C3—C2	125.2 (4)	C25—C26—C27	119.1 (7)
C4—C3—C2	118.5 (4)	C25—C26—H26	120.4
C5—C4—C3	121.3 (5)	C27—C26—H26	120.4
C5—C4—H4	119.3	C26—C27—C28	119.2 (7)
C3—C4—H4	119.3	C26—C27—H27	120.4
C6—C5—C4	120.8 (5)	C28—C27—H27	120.4
C6—C5—H5	119.6	C27—C28—C29	117.8 (7)
C4—C5—H5	119.6	C27—C28—H28	121.1
C5—C6—C7	120.1 (5)	C29—C28—H28	121.1
C5—C6—H6	119.9	N6—C29—C28	123.5 (6)
C7—C6—H6	119.9	N6—C29—H29	118.2
O3—C7—C6	117.1 (5)	C28—C29—H29	118.2
			
O2—Ni2—N1—C1	−1.3 (4)	N4—Ni2—O2—C3	80.9 (4)
O4—Ni2—N1—C1	178.3 (4)	N6—Ni2—O2—C3	−106.9 (4)
N5—Ni2—N1—C1	148.8 (14)	N1—Ni2—O4—C8	−6.2 (3)
N4—Ni2—N1—C1	−91.9 (4)	O2—Ni2—O4—C8	−3.8 (10)
N6—Ni2—N1—C1	89.2 (4)	N5—Ni2—O4—C8	170.5 (3)
O2—Ni2—N1—N2	−172.0 (3)	N4—Ni2—O4—C8	−102.3 (3)
O4—Ni2—N1—N2	7.6 (3)	N6—Ni2—O4—C8	85.6 (3)
N5—Ni2—N1—N2	−21.9 (17)	N2—N1—C1—O1	0.9 (6)
N4—Ni2—N1—N2	97.4 (3)	Ni2—N1—C1—O1	−169.9 (3)
N6—Ni2—N1—N2	−81.5 (3)	N2—N1—C1—C2	−175.6 (4)
C1—N1—N2—C8	180.0 (4)	Ni2—N1—C1—C2	13.7 (7)
Ni2—N1—N2—C8	−7.7 (5)	Ni1—O1—C1—N1	−2.7 (6)
C1—N1—N2—Ni1	1.3 (5)	Ni1—O1—C1—C2	173.8 (3)
Ni2—N1—N2—Ni1	173.68 (17)	N1—C1—C2—C7	164.7 (5)
O1^i^—Ni1—N2—C8	−0.1 (5)	O1—C1—C2—C7	−11.9 (7)
O1—Ni1—N2—C8	179.9 (5)	N1—C1—C2—C3	−13.2 (7)
N2^i^—Ni1—N2—C8	−18 (100)	O1—C1—C2—C3	170.2 (4)
N3^i^—Ni1—N2—C8	89.2 (5)	Ni2—O2—C3—C4	−159.8 (4)
N3—Ni1—N2—C8	−90.8 (5)	Ni2—O2—C3—C2	19.3 (7)
O1^i^—Ni1—N2—N1	178.0 (3)	C7—C2—C3—O2	178.3 (5)
O1—Ni1—N2—N1	−2.0 (3)	C1—C2—C3—O2	−3.8 (8)
N2^i^—Ni1—N2—N1	160 (100)	C7—C2—C3—C4	−2.6 (7)
N3^i^—Ni1—N2—N1	−92.7 (3)	C1—C2—C3—C4	175.3 (5)
N3—Ni1—N2—N1	87.3 (3)	O2—C3—C4—C5	−177.3 (5)
O1^i^—Ni1—N3—C10	−118.7 (5)	C2—C3—C4—C5	3.5 (8)
O1—Ni1—N3—C10	61.3 (5)	C3—C4—C5—C6	−2.2 (9)
N2—Ni1—N3—C10	−16.2 (5)	C4—C5—C6—C7	−0.1 (9)
N2^i^—Ni1—N3—C10	163.8 (5)	C5—C6—C7—O3	−176.4 (6)
N3^i^—Ni1—N3—C10	148 (100)	C5—C6—C7—C2	0.9 (9)
O1^i^—Ni1—N3—C14	57.0 (5)	C3—C2—C7—O3	177.7 (5)
O1—Ni1—N3—C14	−123.0 (5)	C1—C2—C7—O3	−0.4 (8)
N2—Ni1—N3—C14	159.5 (5)	C3—C2—C7—C6	0.5 (8)
N2^i^—Ni1—N3—C14	−20.5 (5)	C1—C2—C7—C6	−177.6 (5)
N3^i^—Ni1—N3—C14	−36 (100)	Ni2—O4—C8—N2	4.1 (6)
N1—Ni2—N4—C15	−63.4 (4)	Ni2—O4—C8—C9	−176.2 (4)
O2—Ni2—N4—C15	−154.1 (4)	N1—N2—C8—O4	2.2 (7)
O4—Ni2—N4—C15	16.0 (4)	Ni1—N2—C8—O4	−179.7 (4)
N5—Ni2—N4—C15	110.8 (4)	N1—N2—C8—C9	−177.5 (4)
N6—Ni2—N4—C15	108.7 (12)	Ni1—N2—C8—C9	0.6 (8)
N1—Ni2—N4—C19	120.4 (4)	C14—N3—C10—C11	−2.3 (11)
O2—Ni2—N4—C19	29.6 (4)	Ni1—N3—C10—C11	173.7 (7)
O4—Ni2—N4—C19	−160.2 (4)	N3—C10—C11—C12	5.3 (15)
N5—Ni2—N4—C19	−65.4 (4)	C10—C11—C12—C13	−4.6 (14)
N6—Ni2—N4—C19	−67.5 (14)	C11—C12—C13—C14	1.7 (14)
N1—Ni2—N5—C20	60.4 (17)	C10—N3—C14—C13	−0.8 (11)
O2—Ni2—N5—C20	−149.7 (5)	Ni1—N3—C14—C13	−176.9 (7)
O4—Ni2—N5—C20	31.3 (5)	C12—C13—C14—N3	1.1 (14)
N4—Ni2—N5—C20	−59.4 (5)	C19—N4—C15—C16	−1.4 (8)
N6—Ni2—N5—C20	120.3 (5)	Ni2—N4—C15—C16	−177.7 (4)
N1—Ni2—N5—C24	−111.6 (15)	N4—C15—C16—C17	2.3 (9)
O2—Ni2—N5—C24	38.3 (5)	C15—C16—C17—C18	−1.8 (9)
O4—Ni2—N5—C24	−140.7 (5)	C16—C17—C18—C19	0.6 (10)
N4—Ni2—N5—C24	128.6 (5)	C15—N4—C19—C18	0.0 (8)
N6—Ni2—N5—C24	−51.7 (5)	Ni2—N4—C19—C18	176.5 (4)
N1—Ni2—N6—C29	−84.5 (5)	C17—C18—C19—N4	0.4 (9)
O2—Ni2—N6—C29	6.3 (5)	C24—N5—C20—C21	0.2 (10)
O4—Ni2—N6—C29	−163.8 (5)	Ni2—N5—C20—C21	−172.2 (5)
N5—Ni2—N6—C29	101.3 (5)	N5—C20—C21—C22	1.4 (12)
N4—Ni2—N6—C29	103.4 (12)	C20—C21—C22—C23	−1.2 (13)
N1—Ni2—N6—C25	105.2 (4)	C21—C22—C23—C24	−0.4 (13)
O2—Ni2—N6—C25	−164.1 (4)	C20—N5—C24—C23	−1.9 (10)
O4—Ni2—N6—C25	25.8 (4)	Ni2—N5—C24—C23	170.5 (5)
N5—Ni2—N6—C25	−69.1 (4)	C22—C23—C24—N5	2.0 (12)
N4—Ni2—N6—C25	−67.0 (14)	C29—N6—C25—C26	0.6 (9)
O1^i^—Ni1—O1—C1	−157 (100)	Ni2—N6—C25—C26	171.4 (5)
N2—Ni1—O1—C1	2.4 (3)	N6—C25—C26—C27	−2.6 (11)
N2^i^—Ni1—O1—C1	−177.6 (3)	C25—C26—C27—C28	2.3 (11)
N3^i^—Ni1—O1—C1	92.3 (3)	C26—C27—C28—C29	−0.2 (12)
N3—Ni1—O1—C1	−87.7 (3)	C25—N6—C29—C28	1.6 (10)
N1—Ni2—O2—C3	−15.3 (4)	Ni2—N6—C29—C28	−169.3 (6)
O4—Ni2—O2—C3	−17.6 (10)	C27—C28—C29—N6	−1.8 (12)
N5—Ni2—O2—C3	168.1 (4)		

Symmetry codes: (i) −*x*, −*y*, −*z*+2.

### Hydrogen-bond geometry (Å, °)

**Table:**

*D*—H···*A*	*D*—H	H···*A*	*D*···*A*	*D*—H···*A*
C27—H27···Cg^ii^	0.93	2.52	3.428 (4)	166

Symmetry codes: (ii) *x*+1, *y*, *z*.
